# Spatial Distribution of Polyphenolic Compounds in Corn Grains (*Zea mays* L. var. *Pioneer*) Studied by Laser Confocal Microscopy and High-Resolution Mass Spectrometry

**DOI:** 10.3390/plants11050630

**Published:** 2022-02-25

**Authors:** Mayya Razgonova, Yulia Zinchenko, Konstantin Pikula, Lyudmila Tekutyeva, Oksana Son, Alexander Zakharenko, Tatiana Kalenik, Kirill Golokhvast

**Affiliations:** 1 Institute of Life Science and Biomedicine, Far Eastern Federal University, 10 Ajax Bay, Russky Island, 690922 Vladivostok, Russia; lat7777@mail.ru (L.T.); oksana_son@bk.ru (O.S.); kalenik.tk@dvfu.ru (T.K.); 2 N.I. Vavilov All-Russian Institute of Plant Genetic Resources, B. Morskaya 42-44, 190000 Saint Petersburg, Russia; yu-zinch@yandex.ru; 3 Polytechnical Institute, Far Eastern Federal University, 10 Ajax Bay, Russky Island, 690922 Vladivostok, Russia; pikula_ks@dvfu.ru (K.P.); golokhvast@sfsca.ru (K.G.); 4 Federal Research Center the Yakut Scientific Center of the Siberian Branch of the Russian Academy of Sciences, 2, Petrovskogo Str., 677000 Yakutsk, Russia; 5 Siberian Federal Scientific Centre of Agrobiotechnology, Centralnaya, Presidium, 633501 Krasnoobsk, Russia; rarf@yandex.ru; 6 Laboratory of Supercritical Fluid Research and Application in Agrobiotechnology, The National Research Tomsk State University, 36, Lenin Avenue, 634050 Tomsk, Russia; 7 Pacific Geographical Institute, Far Eastern Branch of the Russian Academy of Sciences, Radio 7, 690041 Vladivostok, Russia

**Keywords:** confocal microscopy, HPLC–MS/MS, tandem mass spectrometry, polyphenolic compounds

## Abstract

Desirable changes in the biochemical composition of food plants is a key outcome of breeding strategies. The subsequent localization of nutritional phytochemicals in plant tissues gives important information regarding the extent of their synthesis across a tissue. We performed a detailed metabolomic analysis of phytochemical substances of grains from *Zea mays* L. (var. *Pioneer*) by tandem mass spectrometry and localization by confocal microscopy. We found that anthocyanins are located mainly in the aleurone layer of the grain. High-performance liquid chromatography in combination with ion trap tandem mass spectrometry revealed the presence of 56 compounds, including 30 polyphenols. This method allows for effective and rapid analysis of anthocyanins by plotting their distribution in seeds and grains of different plants. This approach will permit a more efficient screening of phenotypic varieties during food plant breeding.

## 1. Introduction

The consumption of corn for 2018–2019 reached 315 million tons in the USA, 276 million tons in China, 63 million tons in the European Union, and 66 million tons in Brazil. In maize breeding, the discovery of genes responsible for the formation of corn endosperm accelerated research on the modeling of nutritional and taste properties of the corn.

The biochemical composition of corn grains, including protein, fatty acid, saccharide, and phenolic content, significantly affect the nutritional quality and taste of corn. The content of essential amino acids, such as valine, isoleucine, leucine, lysine, methionine, threonine, tryptophan, phenylalanine, histidine, and arginine is one of the major factors that determine the nutritional value of corn [1].

Corn grains have the highest polyphenol content (6056.9 mg/kg dry weight or 15.55 μmol/g) among other grains and represent significant interest for phytochemical and metabolomic study [2,3]. Phenolic compounds can have radical scavenging, chelating and antioxidative activity. Polyphenols can prevent oxidative stress caused by metabolic imbalances between the production and scavenging of free radicals [4]. Phenolic compounds can control oxidative stress by neutralizing or reducing the formation of reactive oxygen species (ROS) or restoration of redox homeostasis by strengthening the endogenous defense system or capturing the ROS [5]. The ability of polyphenolic groups to scavenge free radicals is associated with their aromatic rings and a highly conjugated system with many hydroxyl groups. The spatial position and the number of hydroxyl groups are important reference points for the antioxidant activity of phenolic compounds [6].

Fatty acids affect the palatability and especially the odor of foods. In higher plants, the proportion of essential fatty acids in the composition of vegetable fats is very high (up to 90%). It is mainly composed of palmitic, oleic, and linoleic acids. Analysis of the fatty acid composition of corn grains showed the presence of palmitic acid, linoleic acid, vaccenic acid, oleic acid, stearic acid, and eicosanoic acid.

Monosaccharides are derivatives of polyhydric alcohols and serve as a source for the synthesis of disaccharides (sucrose, maltose, lactose), oligosaccharides, and polysaccharides (cellulose and starch). Many of them have a sweet taste, but there are gradations from tasteless to bitter substances that affect the taste qualities of grains, including corn.

Flavonoids act as exogenous antioxidants and are directly oxidized by radicals with the formation of less reactive species through the following mechanisms: inhibition of xanthine oxidase activity, modulation of channel pathways, and inhibition of nitric oxide synthase activity [7]. The antioxidant potential of flavonoids is associated with the location and the total number of OH groups, or rather, with their molecular structure [8]. The use of flavonoids in biological systems holds great promise for bone tissue engineering. Quercetin, an antioxidant flavonoid, when present in the bloodstream, improves vascular health and reduces the risk of cardiovascular disease in its conjugated form. Quercetin and its derivatives prevent blood clotting or thrombosis and prevent the likelihood of stroke [9].

**Figure 1:**
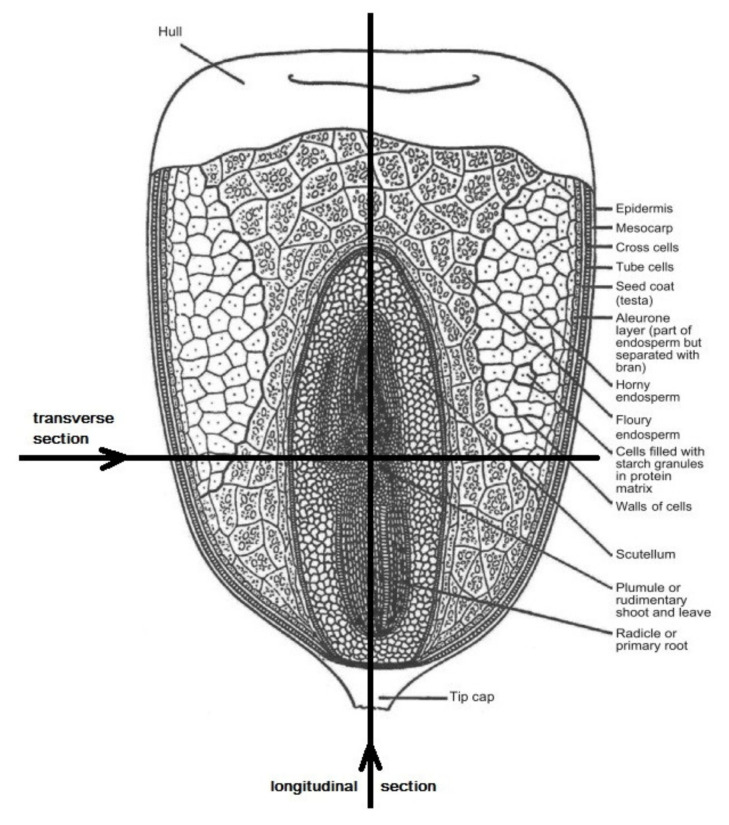
Structure of the grain of dent corn (with the symbolic designation of parts of the grain) (modified from [10]).

The structure of corn grain is presented in Figure 1 [10].

Previous research organized phenolic compounds according to the degree of antioxidant activity: simple phenolic acids < hydroxycinnamic acids < flavonols < flavan-3-ols < dimers of procyanidins [6]. It is known that the antioxidant activity of phenolic acids increases with an increase in the distance separating the carbonyl group and the aromatic ring, and hydroxycinnamic acid derivatives have stronger antioxidant activity than benzoic acid derivatives [11]. The 7,8-double bond of hydroxycinnamic acids also enhances their antioxidant potential, compared with hydroxybenzoic acids.

Jigh-performance liquid chromatography (HPLC) was predominantly used to identify carotenoids [12,13,14] and polyphenols [15,16] in corn grains. A review by Ranilla (2020) summarized the application of metabolomics for the characterization of metabolites in corn grains and emphasized the importance of phenotype–genotype studies aimed to explore corn genetic diversity [17]. The application of electrospray ionization mass spectrometry (ESI–MS) in combination with HPLC is a cost-effective and statistically robust method for high-throughput phenotypic characterization of corn [18]. The HPLC–ESI–MS/MS analytical configuration is widely used for the characterization of phenolic bioactive compounds in worldwide corn biodiversity. Montilla et al. (2011) characterized 10 corn landraces based on the content of phenolic fractions [19]. Das and Singh (2016) characterized four corn hybrids based on the content of phenolic acids, anthocyanins, and flavonols [20].

Another important problem is the study of spatial distribution and composition of phytochemicals in corn grains. Microscopic images are widely used as important sources of information on morphometric characteristics of cells and the architecture of plant tissue [21]. Confocal laser scanning microscopy was previously used to localize the phenolic compounds in different plants [22]. Morphological and biochemical changes in roots of corn *Zea mays* L. were previously studied by confocal microscopy [23,24]. To the best of the authors’ knowledge, no published studies report an application of confocal microscopy for the identification of phytochemicals in the grains of corn *Zea mays* L.

Considering the qualitative data of phytochemical composition obtained by HPLC–MS and literature information regarding the optical properties of identified chemicals, the combination of HPLC data with fluorescence microscopy is a good opportunity to explore the localization of phenolic compounds in plants. The combination of these methods is important for breeding since it allows us to assess whether the genes involved in the synthesis of these substances are expressed only in certain tissues (e.g., the aleurone layer, the germ layer, the vitreous endosperm) or in all grain glutes uniformly. In addition, this approach makes it possible to estimate the number and size of storing organelles (granules, chloroplasts, vesicles), since selection is important in both increasing their number and increasing their size. Thus, the combination of these methods allows us to obtain more complex information about the studied plants.

In this study, we used combined mass spectrometry and confocal laser microscopy to determine the structural properties and phytochemical composition of corn grains. In our case, the combination of HPLC–MS and fluorescence microscopy allowed us to demonstrate the localization of polyphenolic compounds in the grains of corn *Zea mays* L. However, the interpretation of the results of this study requires taking into account the limitations of the study design. The application of combined HPLC and fluorescent microscopy includes the possibility of spatial localization of different groups of plant chemicals in general but not the individual compounds.

## 2. Results

### 2.1. Tandem Mass Spectrometry

**Figure 2:**
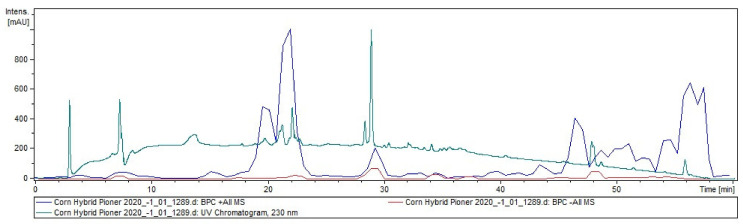
The total compounds chromatogram of *Zea mays* L. (var. *Pioneer*) extract.

The extracts of corn grains were analyzed using liquid chromatography–electrospray ionization mass spectrometry (LC–ESI MS) to explore the diversity of available phytochemicals. The structural identification of each compound was carried out based on their accurate mass and MS/MS fragmentation using LC–ESI MS. In total, 56 compounds were successfully identified and characterized by comparing fragmentation patterns with those available in the literature. The results of a preliminary study showed the presence of 56 compounds corresponding to the genus *Zea*, some of which were identified for the first time in *Zea mays* L. The identified compounds, with molecular formulas *m*/*z* calculated and observed MS/MS data, and their comparative profile for corn grains are summarized in Table A1. The chromatograms of total compounds in the grain extract in positive and negative ionization modes are presented in Figure 2.

In the present study, 30 polyphenol compounds were identified and characterized. In addition, 26 compounds of other classes were identified, including identified for the first time in corn grains oxylipins 13-trihydroxy-octadecenoic acid and 9,12,13-Trihydroxy-*trans*-10-octadecenoic acid.

**Figure 3:**
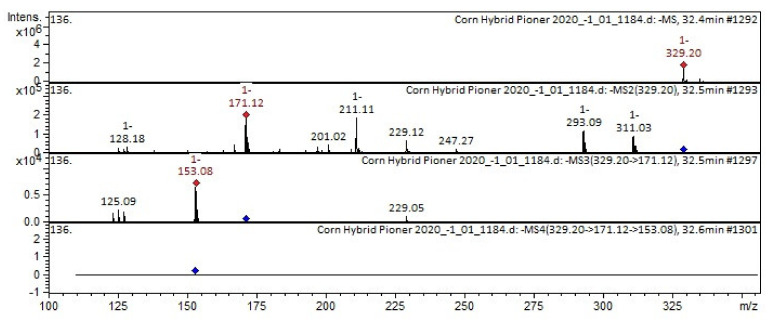
Mass spectrum of 13-trihydroxy-octadecenoic acid (THODE) from the extract of corn grains, *m*/*z* 329.20.

**Figure 4:**
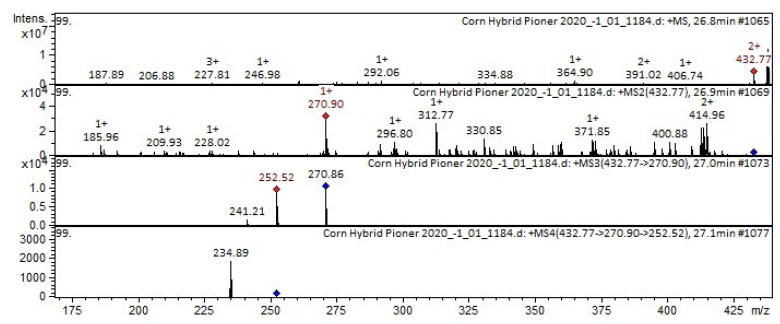
Mass spectrum of pelargonidin-3-*O*-glucoside from extracts of corn grains, *m*/*z* 432.77.

Figure 3 and Figure 4 show examples of the decoding spectra (collision-induced dissociation (CID) spectrum) of the ion chromatogram obtained using tandem mass spectrometry. The [M–H]^−^ ion produced three fragment ions at *m*/*z* 171, *m*/*z* 211, and *m*/*z* 293 (Figure 3). The fragment ion at *m*/*z* 171 yields a daughter ion at *m*/*z* 153. This compound was identified in the bibliography as 13-trihydroxy-octadecenoic acid (THODE) in extracts from *Bituminaria* [25], *Broccoli* [26], *Sasa veitchii* [27].

The mass spectrum in the positive ion mode of pelargonidin-3-*o*-glucoside from extracts of corn grain*s* is shown in Figure 4. The [M + H]^+^ ion produced three fragment ions at *m*/*z* 271, *m*/*z* 415, and *m*/*z* 186 (Figure 4). The fragment ion at *m*/*z* 271 yields two daughter ions at *m*/*z* 253 and *m*/*z* 121. The fragment ion at *m*/*z* 253 yields one daughter ion at *m*/*z* 235. To our knowledge, pelargonidin-3-*o*-glucoside was reported in *Triticum aestivum* L. [28,29], strawberry [30].

### 2.2. Confocal Microscopy

**Figure 5:**
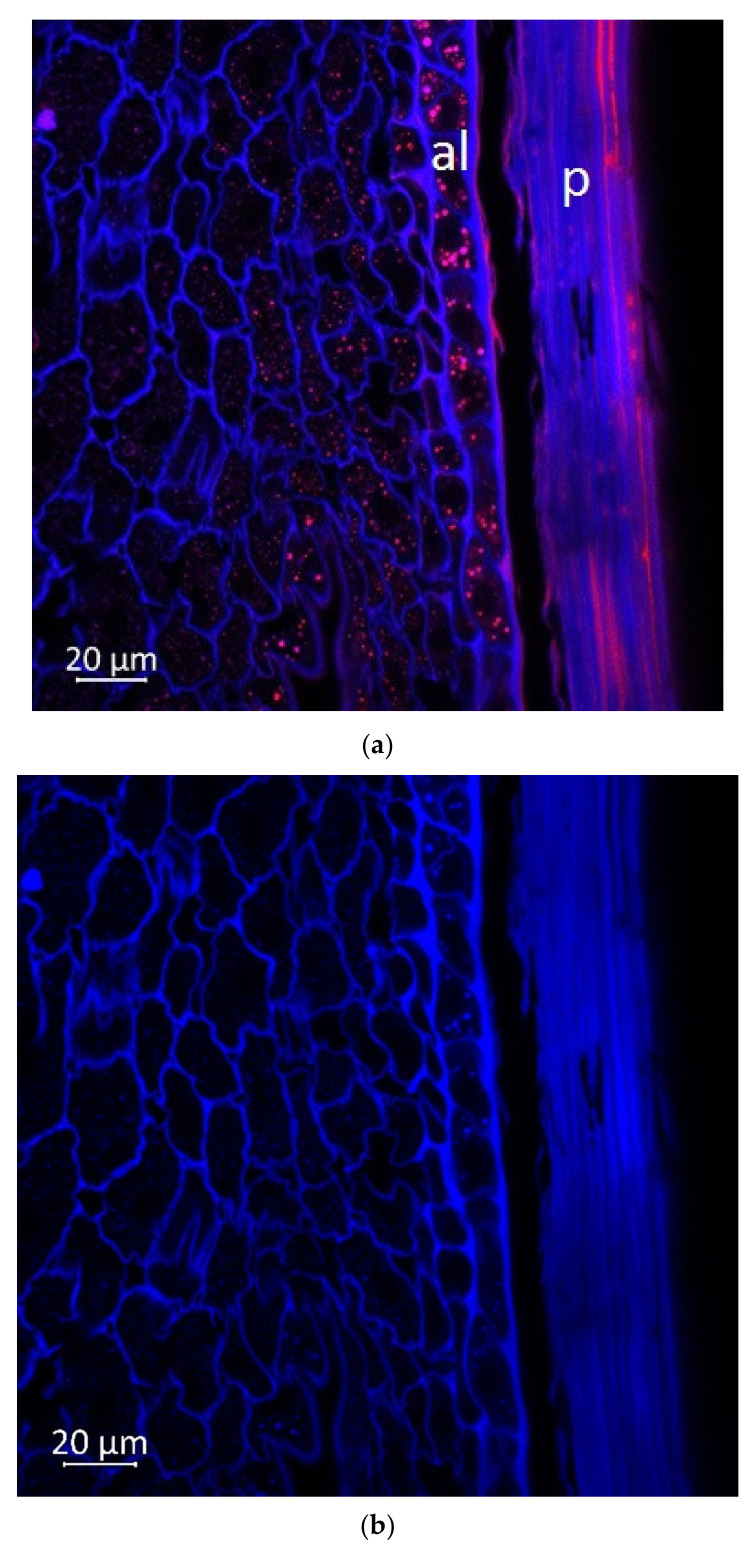

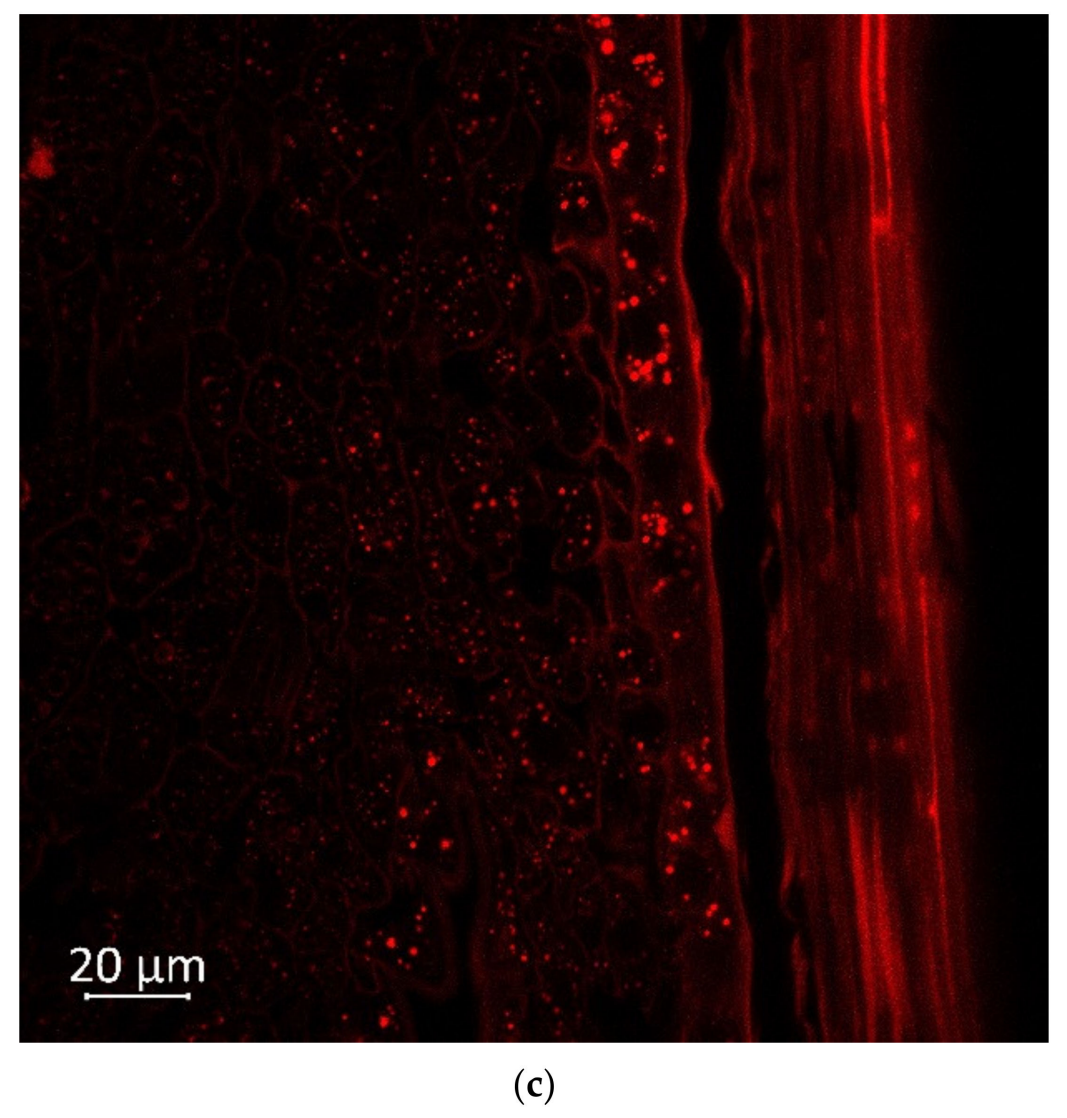
The longitudinal section of the grain (grain margin in the embryo area), 63× magnification: (**a**) multispectral image, excitation 405 nm with the emission in 400–470 nm (blue), excitation 488 nm with the emission in 620–700 nm (red); (**b**) hydroxycinnamic and ferulic acids, and lignin content in the corn grain indicated in blue spectra; (**c**) anthocyanin content in the grain indicated in red spectra; p, pericarp; al, aleurone.

**Figure 6:**
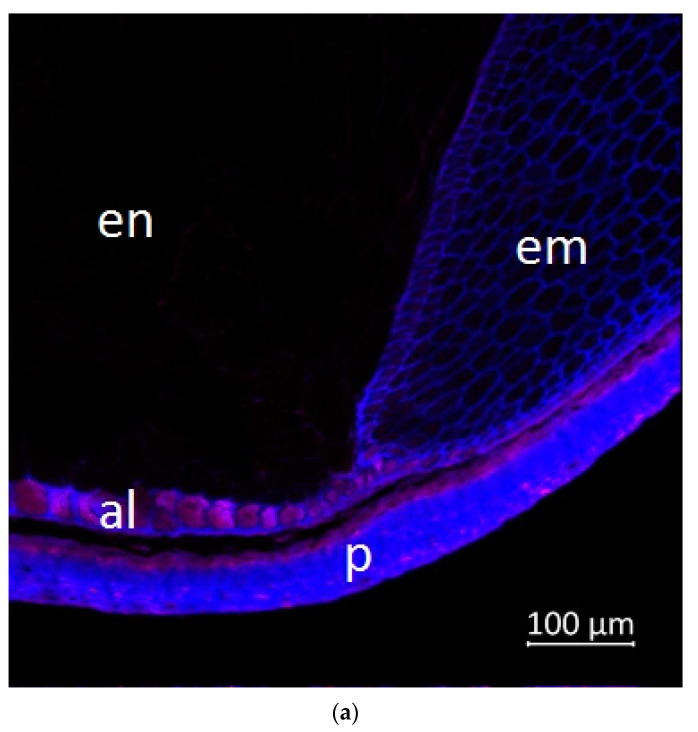

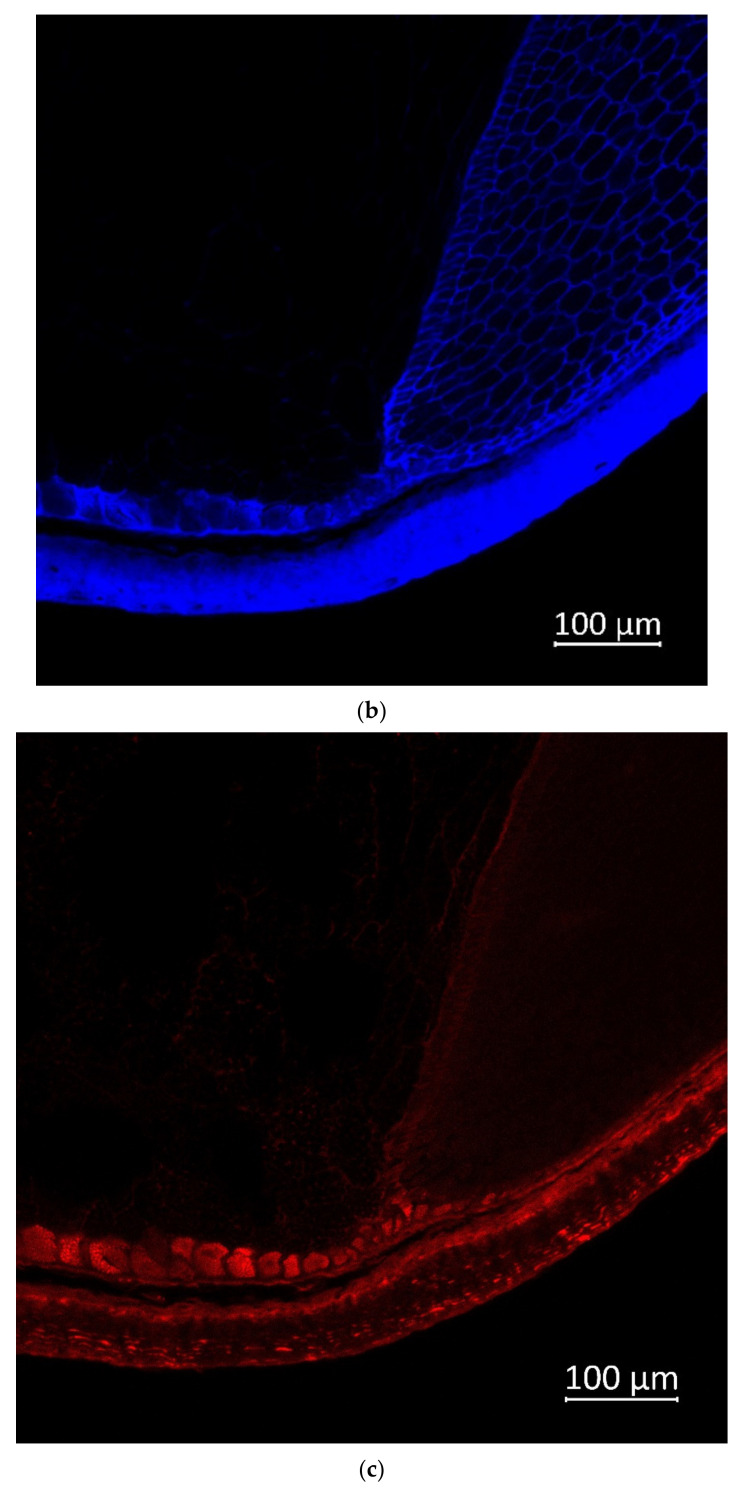
The transverse section of the grain, a border between endosperm (left) and embryo (right), 20× magnification: (**a**) multispectral image, excitation 405 nm with the emission in 400–470 nm (blue), excitation 488 nm with the emission in 620–700 nm (red); (**b**) hydroxycinnamic and ferulic acids, and lignin content in the corn grain indicated in blue spectra; (**c**) anthocyanin content in the grain indicated in red spectra; p, pericarp; al, aleurone; en, endosperm; em, embryo.

**Figure 7:**
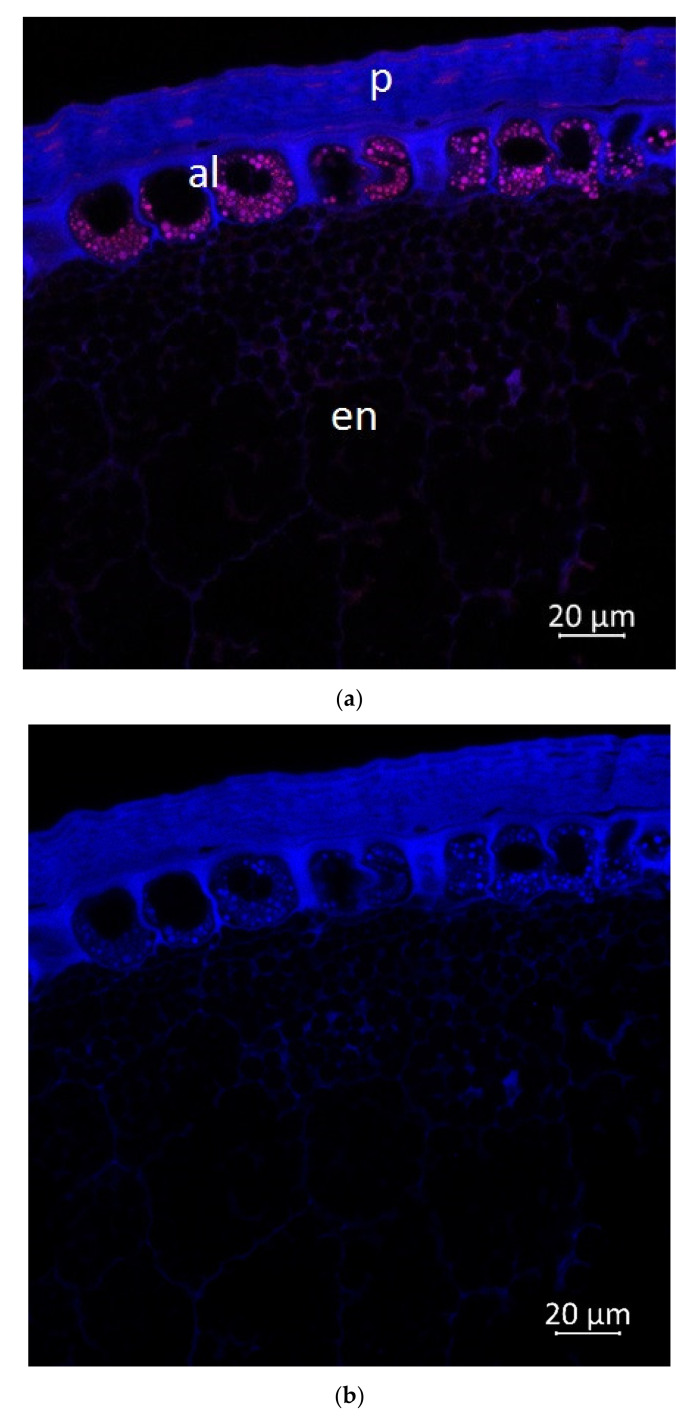

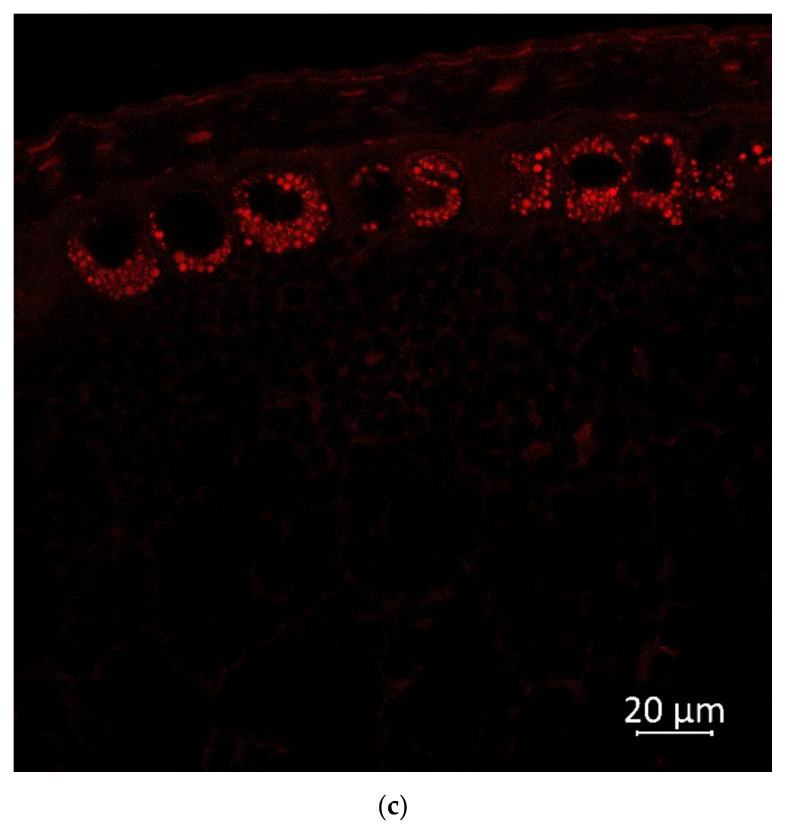
The aleurone layer of the grain (upper margin of the grain, the longitudinal section), 63× magnification: (**a**) multispectral image, excitation 405 nm with the emission in 400–470 nm (blue), excitation 488 nm with the emission in 620–700 nm (red); (**b**) hydroxycinnamic and ferulic acids, and lignin content in the corn grain indicated in blue spectra; (**c**) anthocyanin content in the grain indicated in red spectra; p, pericarp; al, aleurone; en, endosperm.

Confocal microscopy, coupled with Airyscan technology, demonstrated blue (Figure 5b, Figure 6b and Figure 7b) and red fluorescence (Figure 5c, Figure 6c and Figure 7c) in the longitudinal and transverse sections, and in the aleurone layer of the grain, respectively.

According to the literature data, strong blue fluorescence of plant grains under UV excitation could be explained by the presence of phenolic compounds such as hydroxycinnamic [31] or ferulic acid [32], and lignin [33]. The endosperm reveals very low blue autofluorescence (Figure 6 and Figure 7) due to the very low amount of phenolic substances in the endosperm cells of seeds and grains [34]. It was reported that the pericarp of *Zea mays* had a total phenolic content 30–34 fold higher than endosperm [35]. Our results demonstrated that the aleurone cells (Figure 5b, Figure 6b and Figure 7b) and embryo (Figure 7b) were enriched with blue autofluorescence substances. At the same time, it is known that no lignin is present in aleurone [36], but hydroxycinnamic, ferulic, and coumaric acids were reported in aleurone cells of cereals [37,38]. Therefore, the observed blue fluorescence might be caused by hydroxycinnamic, ferulic, and coumaric acids. The main blue fluorescent compound in the pericarp is lignin, which is a heterogeneous mixture of randomly polymerized phenolic monolignols [39].

The emission in the red spectrum mainly occurs due to the presence of various polyphenolic compounds, including anthocyanins and anthocyanidins [40].

## 3. Discussion

It is known that polyphenols have strong antioxidation, anticancer, anti-infection, and other valuable activities [41]. The knowledge of polyphenol distribution in plants will benefit the development of the methods of their direct extraction and further application in the food, pharmaceutical, and cosmetic industries.

Another important problem is the influence of environmental conditions on the polyphenol composition of the plants. The significant genotypic effects and interactions of the genotype with the environment suggest that breeding methodology will require careful site selection and accounting for changes in genotype rank with changes in cultivation sites.

The important characteristics such as grain color, protein, and polyphenol distribution represent significant interest for breeding. In the grain images, the fluorescence signal under UV excitation (405 nm) comes from ferulic acid [42] and lignin [33]. It should be noted that lignin is absent in aleurone, while coumaric and diferulic acids are present in the walls of aleurone cells. These acids can contribute to the autofluorescence of these cell walls [43,44].

Autofluorescence in the aleurone cell walls was not uniform, which is consistent with the studies presented below. Saadi et al. (1998) showed that autofluorescence was more intense in the anticline than in the periclinal cell walls of the corn grains [45]. Moreover, studies have shown that the content of ferulic acid in the anticlinal cell wall of the corn was twice as high as in the periclinal cell wall [46]. However, research by Phillippe et al. [34] argues that anticlinal and periclinal cell walls contain equal amounts of feruloylated arabinoxylan. Therefore, it seems that autofluorescence in the walls of anticlinal aleurone cells can additionally be caused by other substances, for example, coumaric and diferulic acids, which were found in aleurone cells [37].

Our study showed the metabolic profile of the corn *Zea mays* L. (var. *Pioneer*) represented as 56 compounds including 2 compounds identified in corn grains for the first time—namely, oxylipins 13-trihydroxy-octadecenoic acid and 9,12,13-trihydroxy-*trans*-10-octadecenoic acid. Laser microscopy showed the presence of polyphenolic compounds and, in particular, hydroxycinnamic and ferulic acids, and anthocyanins, in the tissues of corn grain.

The method used in this study is effective for rapid analysis of the distribution of polyphenolic compounds in seeds and grains of different plants. This approach allows the study of plant morphology and the characterization of relevant bioactive phytochemicals using an inexpensive and fast methodology. The characterization of novel corn hybrid genotypes harvested from different geographical areas is a strategic problem and addressing this problem would allow sustainable development of local agriculture.

## 4. Materials and Methods

### 4.1. Materials and Chemicals

As an object of research, we used corn grains *Zea mays* L., variety *Pioneer* P1467. The sample was harvested in 2020 in urban-type settlement Kirovsky (Primorsky Krai, Russian Far East) and obtained from a local farmer.

HPLC-grade acetonitrile was purchased from Fisher Scientific (Southborough, UK), MS-grade formic acid was from Sigma-Aldrich (Steinheim, Germany). Ultra-pure water was prepared from SIEMENS ULTRA clear (SIEMENS Water Technologies, Munich, Germany), and all other chemicals were analytical grade.

### 4.2. Fractional Maceration

Fractional maceration technique was applied to obtain highly concentrated extracts [47]. From 500 g of the sample, 4 g of corn seeds was randomly selected for maceration. The total amount of the extractant (ethyl alcohol of reagent grade) was divided into 3 parts, and the grains were consistently infused with the first, second, and third parts. The solid–solvent ratio was 1:20. The infusion of each part of the extractant lasted 7 days at room temperature.

### 4.3. Liquid Chromatography

HPLC was performed using Shimadzu LC-20 Prominence HPLC (Shimadzu, Kyoto, Japan) equipped with a UV sensor and C18 silica reverse phase column (4.6 × 150 mm, particle size: 2.7 µm) to perform the separation of multicomponent mixtures. The gradient elution program with two mobile phases (A, deionized water; B, acetonitrile with formic acid 0.1% *v*/*v*) was as follows: 0–2 min, 0% B; 2–50 min, 0–100% B; control washing 50–60 min 100% B. The entire HPLC analysis was performed with a UV–vis detector SPD-20A (Shimadzu, Kyoto, Japan) at a wavelength of 230 nm for identification of catechin, epicatechin, quercetin, and other compounds [48]; the temperature was 50 °C, and the total flow rate 0.25 mL min^−1^. The injection volume was 10 µL. Additionally, liquid chromatography was combined with a mass spectrometric ion trap to identify compounds.

### 4.4. Mass Spectrometry

MS analysis was performed on an ion trap amaZon SL (Bruker Daltoniks, Bremen, Germany) equipped with an ESI source in negative ion mode. The optimized parameters were obtained as follows: ionization source temperature: 70 °C, gas flow: 9 L/min, nebulizer gas (atomizer): 7.3 psi, capillary voltage: 4500 V, endplate bend voltage: 1500 V, fragmentary: 280 V, collision energy: 60 eV. An ion trap was used in the scan range *m*/*z* 100–1.700 for MS and MS/MS. All experiments were repeated three times. A four-stage ion separation mode (MS/MS mode) was implemented.

### 4.5. Optical Microscopy

Before the microscopic examination, a longitudinal and transverse dissection of corn grains was performed with MS-2 sled microtome (Tochmedpribor, Ukraine). The obtained sliced corn grains were placed on microscopic cover glass through immersion oil to reduce light refraction by air gaps.

The autofluorescence parameters of a slice of corn grain were determined using an inverted confocal microscope (confocal laser scanning microscopy—CLSM, LSM 800, Carl Zeiss Microscopy GmbH, Berlin, Germany). The autofluorescence spectrum was chosen using lambda scan mode of the confocal microscope, which allows to determine the emission maximum in a specific sample and obtain spectral acquisition. The specimen was excited by each laser separately and two main peaks of autofluorescence were revealed: excitation by a UV laser, 405 nm (solid state, diode, 5mW) with the emission maxima in the ranges 400–470 nm (blue); excitation by a blue laser, 488 nm (solid state, diode, 10 mW) with the emission maximum in 620–700 nm (red). The used power and detector gain for blue and red channels were 5% and 750 V, and 7% and 850 V, respectively.

The images were obtained using objectives Plan-Apochromat 20×/0.8 M27 and Plan-Apochromat 63×/1.40 Oil DIC M27 with 20× and 63× magnification, correspondingly. The zoom factor was 0.5. Airyscan at the SR mode was used to increase resolution. The software ZEN 2.1 (Carl Zeiss Microscopy GmbH, Berlin, Germany) was used for image acquisition.

## 5. Conclusions

We determined the qualitative characteristics of secondary metabolites in the tissues of corn *Zea mays* L. (var. *Pioneer)*. In total, 56 compounds were identified, including 2 compounds identified in corn grains for the first time—namely, oxylipins 13-trihydroxy-octadecenoic acid and 9,12,13-trihydroxy-*trans*-10-octadecenoic acid.

The combination of these data with fluorescence microscopy data revealed the most probable localization of phenolic and polyphenolic compounds. In addition, confocal microscopy allowed us to assess the localization of hydroxycinnamic and ferulic acids in aleurone cells and embryos and anthocyanin content in pericarp and aleurone cells. The combination of these methods is important for breeding since it allows us to assess whether the genes involved in the synthesis of these substances are expressed only in certain tissues (the aleurone layer, the germ layer, the vitreous endosperm) or in all grain glutes uniformly. In addition, this approach makes it possible to estimate the number and size of storing organelles (granules, chloroplasts, vesicles), since selection is important both in the area of increasing their number and increasing their size. Thus, the combination of these methods allows us to obtain more complete information about the variables under study. In addition, it shows that confocal microscopy can be used to obtain preliminary information during volumetric screenings of varietal samples, which will allow selecting target groups for more detailed analysis much faster and without the use of expensive reagents.

## Data Availability

The data presented in the current study are available in the article.

## Appendix A

**Table A1:** The list of compounds identified in ethanolic extracts of *Zea mays* L. (var. *Pioneer)* grains.

No.	Class	Compound	Molecular Formula	Calculated Mass	Molecular Ion [M−H]^−^	Molecular Ion [M+H]^+^	Fragmenation Ion MS2	Fragmentation Ion MS3	Fragmentation Ion MS4	References
	POLYPHENOLS									
1	Phenolic acid	Caffeic acid [(2E)-3-(3,4-Dihydroxyphenyl)acrylic acid]	C_9_H_8_O_4_	180.1574		181	135	119		*Dracocephalum palmatum* [49]; *Eucalyptus* [50]; *Triticum* [51]; *Salvia miltiorrhiza* [52]
2	Phenolic acid	Hydroxy methoxy dimethylbenzoic acid	C_10_H_12_O_4_	196.1999		197	177; 153	125		*F. herrerae; F. glaucescens* [53]
3	Phenolic acid	Hydroxyferulic acid	C_10_H_10_O_5_	210.1834		211	193; 125			Andean blueberry [54];
4	Stilbene	Resveratrol [trans-Resveratrol; 3,4′,5-Trihydroxystilbene; Stilbentriol]	C_14_H_12_O_3_	228.2433		229	209	163	146	*A. cordifolia; F. glaucescens; F. herrerae* [53]; *Radix polygoni multiflori* [55]
5	Dihydroxybenzoic acid	3,4-Diacetoxybenzoic acid	C_10_H_11_O_6_	238.1935	237		119			Potato leaves [56]; *Triticum aestivum L.* [57];
6	Flavan-3-ol	Epiafzelechin [(epi)Afzelechin]	C_15_H_14_O_5_	274.2687		275	245; 176	175		*Cassia granidis* [58]; *Cassia abbreviata* [59,60]; *A. cordifolia; F. glaucescens; F. herrerae* [53]
7	Flavonol	Kaempferol [3,5,7-Trihydroxy-2-(4-hydro-xyphenyl)-4H-chromen-4-one]	C_15_H_10_O_6_	286.2363	285		185; 117; 257	117		*Rhus coriaria* (Sumac) [61]; *Lonicera japonicum* [62]; Andean blueberry [54]; Potato [63]; Potato leaves [56];
8	Flavan-3-ol	Catechin [D-Catechol]	C_15_H_14_O_6_	290.2681		291	261; 189	173; 242	191; 143	Potato [64]; *Triticum* [51]; millet grains [65]; *Solanaceae* [66]; Beer [67]; *V. edulis* [53]; *Vigna inguiculata* [68]; *Radix polygoni multiflori* [55]; *Senna singueana* [69]; *Camellia kucha* [70];
9	Flavan-3-ol	(epi)catechin	C_15_H_14_O_6_	290.2681		291	261; 173	243; 173		*C. edulis* [53]; *Radix polygoni multiflori* [55]; *Camellia kucha* [70];
10	Hydroxycinnamic acid	Caffeoylmalic acid	C_13_H_12_O_8_	296.2296	295		277; 171	233; 113		Potato leaves [56]; Strawberry [71]
11	Flavonol	Quercetin	C_15_H_10_O_7_	302.2357		303	275; 245; 203; 175	175		*Rhus coriaria* [61]; Potato leaves [56]; *Vigna sinensis* [72]; *Impatiens glandulifera* Royle [73]; *Eucalyptus* [50]; *Triticum* [51]; millet grains [65]; Tomato [74]; *Bougainvillea* [75]
12	Flavan-3-ol	Gallocatechin [+(−) Gallocatechin]	C_15_H_14_O_7_	306.2675		307	277; 207	207; 159		millet grains [65]; *Solanaceae* [66]; *Licania ridigna* [76]; *G. linguiforme* [53]; *Senna singueana* [69]; *Vaccinium myrtillus* [77]
13	Flavonol	Myricetin	C_15_H_10_O_8_	318.2351		319	291; 219; 174	259; 191	243; 161	*Dracocephalum palmatum* [49]; Potato [63,64]; *Perilla frutescens* [78]; Tomato [74]; Mentha [79]; *Salvia miltiorrhiza* [52]; *Rubus occidentalis* [80]; *Sanguisorba officinalis* [81]; *Radix polygoni multiflori* [55]
14	Flavone	Cirsiliol	C_17_H_14_O_7_	330.2889	329		229; 171; 293	211; 155	183	*Ocimum* [82]
15	Flavone	5,7-Dimetoxy-3,3′,4′-trihydroxyflavone	C_17_H_14_O_7_	330.2889		331	315; 270	313	285; 257	*Oxalis corniculata* [83]
16	Flavone	Luteolin 7,3′-disulphate	C_15_H_10_O_12_S_2_	446.3627		447	287	152		*Zostera marina* [84]
17	Flavone	Apigenin 7-sulfate	C_15_H_10_O_8_S	350.3001		351	337; 308	308; 291		*G. linguiforme* [53]; sulphates [85]
18	Lignan	Matairesinol [(−)-Matairesinol; Artigenin Congener]	C_20_H_22_O_6_	358.3851		359	324; 289; 127	144	127	*Punica granatum* [86]; Wheat [87]; Lignans [88]
19	Hydroxycinnamic acid derivative	Caffeic acid derivative	C_16_H_18_O_9_Na	377.2985	377		341; 215	179; 113		Bougainvillea [75]
20	Gallate ester, derivative of epiafzelechin	Epiafzelechin 3-*O*-gallate	C_22_H_18_O_9_	426.3729		427	301; 171; 382	171		*Camellia kucha* [70];
21	Flavone	Apigenin-*C*-hexoside	C_21_H_20_O_10_	432.3775		433	418; 314; 265; 219; 155	257; 169		*Triticum durum* [89]; Beer [67]
22	Anthocyanidin	Pelargonidin-3-*O*-glucoside (callistephin)	C_21_H_21_O_10_	433.3854		433	271; 185	253; 121	235	*Triticum aestivum L.* [28,29]; strawberry [30]
23	Anthocyanidin	Cyanidin-3-*O*-glucoside [Cyanidin 3-*O*-beta-D-Glucoside; Kuromarin]	C_21_H_21_O_11_+	449.3848	447		285	199		*Triticum* [29,51]; acerola [90]; rice [91]; *Vigna sinensis* [72]; Rapeseed petals [92]
24	Flavone	Luteolin-7-*O*-beta-glucuronide	C_21_H_18_O_12_	462.3604		463	447; 395; 359; 285; 199; 149	287; 199		*Mentha* [93,94]; rat plasma [95]; *Newbouldia laevis* [60]
25	Flavonol	Kaempferol-3-*O*-glucuronide	C_21_H_18_O_12_	462.3604		463	287; 198	269; 198		Strawberry [71]; *A.cordifolia; G. linguiforme* [53]; *Rhus coriaria* [61]
26	Anthocyanidin	Delphinidin malonyl hexoside	C_24_H_23_O_15_	551.4304		551	465; 287; 185	287; 115		*F. glaucescens* [53]
27	Flavone	Chrysoeriol *C*-hexoside-*C*-pentoside	C_27_H_30_O_15_	594.5181		595	578; 536; 509; 425	294		*Triticum aestivum* L. [57,96]; *T. durum* [89]
28	Flavonol	Quercetin 3,4′-di-*O*-beta-glucopyranoside [Quercetin diglucoside]	C_27_H_30_O_17_	626.5169		627	465	447; 405; 303		Potato leaves [56]; Potato [63]; Rapeseed petals [92];
29	Flavone	Tricin trimethyl ether 7-*O*-hexosyl-hexoside	C_30_H_36_O_17_	668.5966		669	345; 387; 283			*Triticum aestivum L.* [97]
30	Flavan-3-ol	(Epi)fisetinidol-(epi)catechin-A-(epi)fisetinidol	C_45_H_36_O_16_	832.7577	831		721; 693; 609; 575; 537; 506			*Chamaecrista nictitans* [98]
	OTHER COMPOUNDS									
31	Amino acid	L-Lysine	C_6_H_14_N_2_O_2_	146.1876		147	119			*Lonicera japonica* [62];
32	Amino acid	L-threanine	C_7_H_14_N_2_O_3_	174.1977		175	159			*Camellia kucha* [70]
33	Amino acid	L-Tryptophan [Tryptophan; (S)-Tryptophan]	C_11_H_12_N_2_O_2_	204.2252		205	161; 159	143		*Passiflora incarnata* [99]; *Vigna unguiculata* [100]; *Camellia kucha* [70];
34	Omega-5 fatty acid	Myristoleic acid [Cis-9-Tetradecanoic acid]	C_14_H_26_O_2_	226.3550		227	209; 168	127		*F. glaucescens* [53]
35	Monobasic saturated carboxylic acid	Myristic acid [Tetradecanoic acid; N-Tetradecanoic acid]	C_14_H_28_O_2_	228.3709		229	142; 205	114		*Rhododendron adamsii* [101]
36	Medium-chain fatty acid	Hydroxy dodecanoic acid	C_12_H_22_O_5_	246.3001		247	238	203	174	*F. glaucescens* [53]
37	Ribonucleoside composite of adenine (purine)	Adenosine	C_10_H_13_N_5_O_4_	267.2413		268	136			*Lonicera japonica* [62]
38	Omega-3 fatty acid; octadecatetraenoic acid	Stearidonic acid [6,9,12,15-Octadecatetraenoic acid; Moroctic acid]	C_18_H_28_O_2_	276.4137		277	259; 177	177		*Salviae Miltiorrhizae* [102]; *G. linguiforme* [53]; *Rhus coriaria* [61]
39	Omega-3 fatty acid	Linolenic acid (Alpha-Linolenic acid; Linolenate)	C_18_H_30_O_2_	278.4296		279	243; 173	173	131	*Salviae* [102]; rice [91]; *Pinus silvestris* [103]
40	Diterpenoid	Isocryptotanshinone II	C_19_H_20_O_3_	296.3603		297	279; 197	173		*Salviae Miltiorrhizae* [102]
41	Alpha-omega dicarboxylic acid	Octadecanedioic acid [1,16-Hexadecanedicarboxylic acid]	C_18_H_34_O_4_	314.4602	313		295; 183	293; 179	275; 177	*F. glaucescens* [53]
42	Unsaturated essential fatty acid	Oxo-eicosatetraenoic acid	C_20_H_30_O_3_	318.4504		319	301	186		*F. potsii* [53]
43	Oxylipin	13- Trihydroxy-Octadecenoic acid [THODE]	C_18_H_34_O_5_	330.4596	329		171; 211; 293	153		*Bituminaria* [25]; *Broccoli* [26]; Sasa veitchii [27]
44	Oxylipin	9,12,13- Trihydroxy-*trans*-10-octadecenoic acid	C_18_H_34_O_5_	330.4596	329		171; 229	127		Potato leaves [56]
45	Unsaturated essential fatty acid	Eicosatetraenedioic acid	C_20_H_30_O_4_	334.4498		335	321; 124	291		*G. linguiforme* [53]
46	Isoquinoline alkaloid	Berberine [Berberin; Umbelletine; Berbericine]	C_20_H_18_NO_4_	336.3612		337	321; 225	291	291	*Tinospora cordifolia* [104,105]
47	Pentacyclic diterpenoid	Gibberellic acid	C_19_H_22_O_6_	346.3744		347	345; 259	329; 173	289	*Triticum aestivum* [106]
48	Berberine alkaloid	Palmatine [Berbericinine; Burasaine]	C_21_H_22_NO_4_	352.4037		353	337; 163	308	293	*Ocotea* [107,108]
49	Androgen; anabolic steroid	Vebonol	C_30_H_44_O_3_	452.6686		453	435; 336; 209	336; 226	209	*Rhus coriaria* [61]; *Hylosereus polyrhizus* [109]
50	Triterpenoid	Oleanoic acid	C_30_H_48_O_3_	456.7003		457	411; 249; 183	227; 169		Pear [110]; *Ocimum* [82]
51	Triterpenoid	Maslinic acid	C_30_H_48_O_4_	472.6997		473	425; 319; 201	291		*Pear* [110]; *Folium Eriobotryae* [111]; *Malus domestica* [112]
52	Thromboxane receptor antagonist	Vapiprost	C_30_H_39_NO_4_	477.6350		478	460; 337; 263; 155	263; 155	245; 189; 111	*Rhus coriaria* [61]; *Hylosereus polyrhizus* [109]
53	Indole sesquiterpene alkaloid	Sespendole	C_33_H_45_NO_4_	519.7147		520	184	184; 125		*Rhus coriaria* [61]; *Hylosereus polyrhizus* [109]
54	2-arylbenzofuran flavonoid	Lithospermic acid A	C_27_H_22_O_12_	538.4564		539	521; 409; 340; 241	395; 252; 167		*Mentha* [79,93,94]; *Salvia multiorrizae* [52]
55	Carotenoid	(all-E)-lutein 3′-*O*-myristate	C_40_H_54_O	550.8562		551	533; 505; 469;429; 373; 345	453; 410		Carotenoids [113]
56	Triterpenoid	3-*O*-glucuronide-29-hydroxyoleanolic acid	C_35_H_52_O_11_	648.7808		649	473; 367; 291; 229	456; 385; 269	408; 305; 262; 187	Bougainvillea [75]
